# Cost analysis of nurse-lead telephone follow-ups after benign hysterectomy: a randomized, single-blinded, four-arm, controlled multicenter trial

**DOI:** 10.1007/s00404-025-08035-1

**Published:** 2025-05-02

**Authors:** Gulnara Kassymova, Thomas Davidson, Gunilla Sydsjö, Ninnie Borendal Wodlin, Lena Nilsson, Preben Kjølhede

**Affiliations:** 1 Department of Obstetrics and Gynecology in Linköping, and Department of Biomedical and Clinical Sciences, Linköping University, 58245 Linköping, Sweden; 2 Department of Health, Medicine and Caring Sciences, Linköping University, Linköping, Sweden; 3 Department of Anesthesiology and Intensive Care in Linköping and Department of Biomedical and Clinical Sciences, Linköping University, Linköping, Sweden

**Keywords:** Hysterectomy, Telephone follow-up, Cost minimization analysis, Healthcare, Patient-centered care

## Abstract

**Purpose:**

The aim of the study was to evaluate the health economics of nurse-led telephone follow-up contacts (TFUs) within six weeks after benign hysterectomy in a societal perspective, using a cost minimization analysis model.

**Methods:**

A randomized, single-blinded, controlled, Swedish multicenter study comprising 487 women undergoing benign hysterectomy. The women were allocated 1:1:1:1 to either Group A (no TFUs), Group B (one clinically structured TFU the day after discharge), Group C (as B, but with additional TFUs once weekly for six weeks, in total six TFUs), or Group D (as C, but by applying a coaching technique). Time consumption for planned TFUs, informal care, and the number of unplanned telephone contacts and visits were recorded. Costs were assessed using a cost-per-patient price list for Linköping University Hospital.

**Results:**

The total cost per patient more than doubled in the groups with repeated TFUs (Groups C and D) compared with no TFUs (Group A). Group D demonstrated fewer unplanned telephone contacts and lower informal care costs. Group B, with only one TFU, exhibited the highest time consumption for TFU. The additional costs of six TFUs, with or without coaching, substantially increased the costs. The coaching TFU group (Group D) had the lowest cost for informal care.

**Conclusion:**

TFUs appeared to be costly and an inefficient way of using healthcare resources after benign hysterectomy. The coaching TFU seemed to reduce unplanned telephone contacts and lower informal care costs. Careful consideration of the costs and the impact on clinical outcomes is important before implementing TFU after surgery.

**Trial registration:**

This study is registered retrospectively in ClinicalTrial.gov: NCT01526668 on January 27, 2012. Date of enrollment of first patient: October 11; 2011.

## What does this study add to the clinical work

**Table:** 

Although coaching TFUs seemed to reduce unplanned telephone contacts and lower informal care costs, TFUs appeared to be a costly and inefficient way of using healthcare resources after benign hysterectomy. Careful consideration of the costs and the impact on clinical outcomes is important before implementing TFU after surgery.	

## Introduction

Healthcare in Sweden is tax-funded and consequently has limited resources. Thus, the financial resources need to be managed wisely to achieve a high intervention efficiency while minimizing the costs, without compromising the quality and safety of care.

Health economic evaluations are, among other things, used to help decision-makers deal with resource allocation and healthcare planning. By comparing the costs and effects of different interventions, policymakers and healthcare providers can make more informed decisions on how to allocate resources and improve health outcomes for patients and society [1].

Hysterectomy is one of the most common major gynecological procedures performed worldwide. Hysterectomy on benign indications is generally considered cost effective as it improves health-related quality of life at an acceptable cost. However, it is difficult to draw overall conclusions on the cost-effectiveness of benign hysterectomy due to heterogeneity and methodological differences in studies addressing this topic [2].

An important issue to take into consideration concerning cost-effectiveness in surgery is the impact of postoperative recovery. The Enhanced Recovery After Surgery (ERAS) pathways are approaches to optimizing the pre- and perioperative care [3, 4]. In ERAS settings, the overall hospital costs decreased after hysterectomy due to shorter hospitalization without increasing complication or readmission rates [5]. The patients’ experience of ERAS programs in qualitative studies indicates a desire to extend the program with contact from professional or experienced patient volunteers following hospital discharge, to offer support and guidance [6, 7]. Shortening of hospital stay can therefore also be an incentive to enhance the post-discharge support to speed up recovery. However, the literature is sparse on information of this issue, and there are no validated follow-up programs, guidelines, or treatment models to handle postoperative recovery. Based on healthcare traditions and the women’s preferences, different methods of follow-up contact are applied after benign hysterectomy. One of these methods is nurse-led telephone follow-up (TFU) contact after discharge from the hospital.

The current evidence on the costs and cost-effectiveness of TFU after benign hysterectomy is scanty and heterogeneous [8]. Thus, it remains to be seen if TFU is cost effective. However, telephone-based health coaching for chronic diseases seemed to be a cost-effective intervention from a one-year perspective but with a substantial variation across patient groups [9]. Thus, applying a coaching model to the TFU may perhaps increase the possibility of achieving a cost-effective result of recovery also after benign hysterectomy.

The implementation of TFUs after hysterectomy in Sweden has been debated. We have recently shown that TFUs, also including coaching, did not improve recovery after benign hysterectomy concerning health-related quality of life (HRQoL), duration of sick leave, intensity of postoperative symptoms, or analgesics consumption [10, 11]. Since no effect was seen in the recovery of HRQoL between different TFU models, it seemed inappropriate to analyze the cost-effectiveness of TFUs. Instead, a cost minimization analysis (CMA) could be appropriate. A CMA determines the least costly intervention when the outcomes of the interventions are equal. It involves identification, quantification, and valuing of the costs in monetary terms of at least two alternative strategies [12].

This study presents a secondary outcome of a randomized, single-blinded, four-arm, controlled multicenter study, the Post-hysterectomy Recovery trial (POSTHYSTREC) [10, 11]. The aim was to perform a CMA of four TFU models, from a societal perspective, of women undergoing benign hysterectomy, within six weeks after their discharge.

## Materials and methods

The POSTHYSTREC, a randomized single-blinded, four-arm, controlled multicenter intervention trial, was conducted at five departments of obstetrics and gynecology in five public hospitals in the southeast health region of Sweden between October 2011 and May 2017. This study was performed in line with the principles of the Declaration of Helsinki. Ethical approval was obtained from the Regional Ethical Board at Linköping University (Dnr.2011/106-31, approval date 23 May; 2011).

### Study population

The study population, flow chart, the inclusion/exclusion criteria, the randomization process, the interventions, and surgery have previously been described in detail [10]. Briefly, women admitted to the hospitals for benign hysterectomy were eligible for this study. The main inclusion criteria were age between 18 and 60 years, speaking fluent Swedish, and having access to a private telephone or the internet. Exclusion criteria were genital prolapse as an indication for the hysterectomy, physical or mental disability, severe psychiatric disease, and current drug or alcohol abuse, previous oophorectomy or the present operation would leave the woman without ovaries. After receiving written and oral information and having signed the consent document approximately one week before surgery, the participants were randomized into the ratio 1:1:1:1 to one of four TFU models. The outcome of the allocation was kept secret from the participant until the moment of discharge from hospital and only information about the TFU model that should be used for the specific participant was given. Thus, the women were blinded to the other interventions.

### TFU intervention models

- Group A—no planned follow-up contacts with the healthcare services after discharge. The patient was requested to contact the healthcare services, if necessary.
- Group B—one planned, ordinary clinically structured TFU with the research nurse (RN) the day after discharge. Thereafter, the patient was requested to contact the healthcare services, if necessary.
- Group C—planned, ordinary clinically structured TFU with the RN the day after discharge and then once weekly for six weeks.
- Group D—planned, structured coaching TFU with the RN the day after discharge, and then once weekly for six weeks.

The content of the TFU models and the orientation of the coaching model have previously been described in detail [10, 11].

### Data collection

Demographic and clinical data were collected prospectively. The participants filled in two generic HRQoL forms, the EQ-5D-3L [13, 14] and the SF-36 [15, 16], preoperatively and six weeks after the hysterectomy. The RN met all participants six weeks after the hysterectomy at the end of the study for collection of the study-specific forms and diaries, and for an interview. All readmissions and reoperations were registered. The RN registered the time consumption of the planned TFUs and the number and time consumption of unplanned telephone contacts (uTCs) along with the healthcare facility of unplanned visits (uVs) (hospital outpatient facility or primary healthcare) and the healthcare provider (gynecologist, physician, or nurse).

The participants were requested to report in a diary, week-by-week, if and how many hours per week they had informal care from a family member, partner, friend, or neighbor after discharge due to disability caused by the hysterectomy.

### Determination of costs

The calculations of costs followed the structure of a cost analysis [1] and the CHEERS 2022 requirements [17]. A societal perspective was used, capturing both direct healthcare costs, productivity losses, and costs due to informal care. The calculation of hospital costs was based on the CPP principles [18]. CPP is a method to estimate healthcare costs per care contact and patient. Thus, medical data and costs are linked to an individual patient. In this study, the 2022 CPP list from the University Hospital in Linköping was used. The CPPs relevant to this study have been extracted from the list and are presented in Table 1 as € in rounded values. The average exchange rate for 2022 was 1 € = 1.0501 US$ = 10.6317 Swedish kronor [19].

**Table 1:** List for hospital costs per patient according to the CPP list, version 2022, from the University Hospital in Linköping [13]

Entity	Basis of price	Price in €
Telephone contact or visit with nurse	Standard price per contact or visit	76
Visit to gynecologist in hospital	Standard price per visit	133
Visit to physician in outpatient care	Standard price per visit	219
Informal care cost	Price per hour	7.3

Average exchange rate 2022: 1 € = 1.0501 US$ = 10.6317 Swedish kronor

Direct cost covers all in-hospital costs and post-discharge costs. The latter included all planned and unplanned contacts. The indirect costs, that is the societal costs, as a measure of loss of production, were based on sick leave duration and information on the maximal level of compensation provided by the Social Insurance Agency for 2021 along with the costs for informal care. The costs of informal care were estimated by multiplying the mean number of hours spent by the mean national hourly gross wage, including employer and social security contributions [20, 21]. Leisure time was valued at 35% of the gross wage rate. In 2022, this value was about 7.3 € per hour.

### Statistics

The software TIBCO Statistica^®^ v13.5.0 (TIBCO Software Inc. 3307 Hillview Avenue, Palo Alto, CA 94304 USA) was used to process the data. Data are presented as mean and standard deviation (SD), median and interquartile range (IQR), or number and percent, as appropriate. Nominal data were analyzed by means of Pearson’s Chi-squared test. Continuous, normally distributed data were analyzed using one-way analysis of variance (ANOVA), and not normally distributed data were evaluated by means of Mann–Whitney *U*-test or Kruskal–Wallis ANOVA, as appropriate. The subsequent post hoc test between-group differences were conducted using multiple comparisons of mean ranks for all groups. The level of significance was set at *p* < 0.05 (two-sided testing).

## Results

Out of 525 women enrolled in this study, 487 completed this study. Baseline demographic and clinical outcomes including preoperative and six-week assessment of HRQoL outcomes in relation to intervention group are presented in Table 2.

**Table 2:** Demographic and clinical data of 487 women undergoing benign hysterectomy in relation to the intervention group

	Group A (*n* = 120)	Group B (*n* = 122)	Group C (*n* = 125)	Group D (*n* = 120)	*p*-value
Age (years)	45.5 (5.3)	47.2 (5.6)	46.2 (5.3)	47.0 (5.8)	0.08^a^
BMI (kg/m^2^)	26.8 (4.8)	27.0 (4.8)	26.7 (4.6)	26.5 (4.6)	0.85^a^
Smoking	18 (15.5%)	9 (7.6%)	18 (14.4%)	11 (9.6%)	0.18^b^
Gainfully employment	107 (89.2%)	117 (95.9%)	111 (88.8%)	113 (94.2%)	0.10^b^
Comorbidity					
Mental illness	23 (19.2%)	8 (6.6%)	20 (16.0%)	14 (11.7%)	0.02^b^
Chronic pain disorder	28 (23.3%)	30 (24.6%)	29 (23.2%)	31 (25.8%)	0.96^b^
Hysterectomy indication					
Myoma uteri	58 (48.3%)	65 (53.3%)	47 (37.6%)	53 (44.2%)	0.37^b^
Bleeding disorder	32 (26.7%)	23 (18.8%)	35 (28.0%)	35 (29.2%)	
Myoma and bleeding	10 (8.3%)	14 (11.5%)	21 (16.8%)	13 (10.8%)	
Cervical dysplasia	14 (11.7%)	12 (9.8%)	14 (11.2%)	9 (7.5%)	
Pain	5 (4.2%)	8 (6.6%)	8 (6.4%)	9 (7.5%)	
Others	1 (0.8%)	0 (0.0%)	0 (0.0%)	1 (0.8%)	
ASA classification					
Class 1	84 (70.0%)	78 (63.9%)	79 (63.2%)	79 (65.8%)	0.37^b^
Class 2	35 (29.2%)	40 (32.8%)	39 (31.2%)	39 (32.5%)	
Class 3	1 (0.8%)	4 (3.3%)	7 (5.6%)	2 (1.7%)	
Mode of hysterectomy					
Abdominal	97 (80.8%)	98 (80.3%	98 (78.4%)	90 (75.0%)	0.68^b^
Vaginal	23 (19.2%)	24 (19.7%)	27 (21.6%)	30 (25.0%)	
Clavien–Dindo complication grading^d^					
Grade 1	17 (14.2%)	15 (12.3%)	16 (12.8%)	9 (7.5%)	0.64^b^
Grade 2	17 (14.2%)	19 (15.6%)	24 (19.2%)	16 (13.3%)	
Grade 3	3 (2.5%)	6 (4.9%)	4 (3.2%)	3 (2.5%)	
Readmission within six weeks postoperatively	3 (2.5%)	7 (5.7%)	6 (4.8%)	8 (6.7%)	0.48^b^
Sick leave duration (days)	26.8 (10.4)	28.1 (10.7)	28.0 (10.0)	26.9 (10.8)	0.71^c^
EQ-5D-3L health index					
Preoperatively	0.79 (0.22)	0.79 (0.21)	0.79 (0.22)	0.80 (0.18)	0.99^c^
Six weeks postoperatively	0.91 (0.14)	0.89 (0.17)	0.89 (0.19)	0.89 (0.17)	0.90^c^
SF-36					
PCS preoperatively	46.9 (10.6)	47.1 (9.9)	48.1 (9.1)	48.0 (8.5)	0.68^c^
MCS preoperatively	47.2 (10.7)	46.4 (10.3)	47.0 (11.1)	48.0 (10.1)	0.70^c^
PCS six weeks postoperatively	39.4 (7.8)	40.3 (9.3)	41.9 (8.3)	40.5 (8.8)	0.11^c^
MCS six weeks postoperatively	47.6 (10.2)	47.8 (10.6)	46.9 (12.2	49.4 (11.4)	0.17^c^

Figures denote mean and (standard deviation) or number of women and (percent)

*EQ-5D-3L* the EuroQol Group five dimensions with three level form; *MCS* mental component summary; *PCS* physical component summary; *SF-36* Short-Form Health Survey

^a^One-way ANOVA; ^b^Pearson’s Chi-squared test; ^c^Kruskal–Wallis ANOVA; ^d^Complications within six weeks postoperatively

The data of the TFUs, uTCs, uVs, and informal care are shown in Table 3. Group C and D had the same number of TFUs but differed in the content of the TFU. In Group C, the nurse applied a traditional clinically structured counseling technique, whereas a coaching technique was used in Group D. The numbers of uTCs, uVs, and the consumption of informal care were similar in Groups A, B and C, whereas Group D had a substantially lower number of uTCs and time consumption of informal care.

**Table 3:** Summary report of TFUs, uTCs, uVs, and informal care in relation to the intervention group

	Group A (*n* = 120)	Group B (*n* = 122)	Group C (*n* = 125)	Group D (*n* = 120)	*p*-value
Time consumption of TFU 1 (minutes), mean (SD)	NA	9.8 (4.5)	8.3 (3.8)	8.7 (4.3)	0.02^a^
Time consumption of TFU 2 (minutes), mean (SD)	NA	NA	7.4 (4.0)	8.5 (4.1)	< 0.01^b^
Time consumption of TFU 3 (minutes), mean (SD)	NA	NA	7.5 (3.9)	7.7 (3.8)	0.82^b^
Time consumption of TFU 4 (minutes), mean (SD)	NA	NA	6.7 (3.4)	7.0 (3.6)	0.97^b^
Time consumption of TFU 5 (minutes), mean (SD)	NA	NA	6.3 (2.8)	6.3 (2.7)	0.81^b^
Time consumption of TFU 6 (minutes), mean (SD)	NA	NA	5.5 (3.0)	5.6 (2.9)	0.66^b^
Summary time consumption (minutes), mean (SD)	NA	9.8 (4.5)	41.6 (15.5)	43.8 (16.3)	0.39^b,c^
Number of women with uTCs	57 (47.5%)	65 (53.3%)	62 (49.6%)	40 (33.3%)	0.01^d^
Number of uTCs					
Total number	110	135	109	73	
Mean (SD)^e^	0.9 (1.4)	1.1 (1.5)	0.9 (1.2)	0.6 (1.2)	0.01^a^
Median (IQR)^e^	0.0 (0.0–1.0)	1.1 (0.0–2.0)	0.0 (0.0–1.0)	0.6 (0.0–1.0)	
Median (IQR)^f^	1.0 (1.0–2.0)	1.0 (1.0–3.0)	1.0 (1.0–2.0)	1.0 (1.0–2.0)	0.71^a^
Time consumption uTCs (minutes)					
Mean (SD)	5.4 (9.1)	6.9 (11.0)	5.3 (7.8)	3.2 (7.3)	< 0.01^a^
Median (IQR)	0.0 (0.0–7.0)	4.0 (0.0–10.0)	0.0 (0.0–8.0)	0.0 (0.0–5.0)	
Number of women with uVs^g^	57 (47.5%)	54 (44.3%)	62 (49.6%)	45 (37.5%)	0.25^d^
In primary healthcare	21 (17.5%)	22 (18.0%)	23 (18.4%)	12 (10.0%)	0.23^d^
In hospital outpatient facility	41 (34.2%)	43 (35.2%)	52 (41.6%)	38 (31.7%)	0.41^d^
Number of uVs^g^					
Total number/median (IQR)	97/0.0 (0.0–1.0)	143/0.0 (0.0–1.0)	153/1.0 (0.0–2.0)	113/0.0 (0.0–1.0)	0.34^a^
In primary healthcare					
Total number/median (IQR)	28/0.0 (0.0–0.0)	66/0.0 (0.0–0.0)	48/0.0 (0.0–0.0)	25/0.0 (0.0–0.0)	0.22^a^
In hospital outpatient facility					
Total number/median (IQR)	69/0.0 (0.0–1.0)	77/0.0 (0.0–1.0)	105/0.0 (0.0–1.0)	88/0.0 (0.0–1.0)	0.43^a^
Informal care, week 1 (hours), median (IQR)	5.0 (0.0–14.3)	6.0 (0.5–14.0)	6.0 (0.0–12.0)	4.0 (0.3–9.5)	0.32^a^
Informal care, week 2 (hours), median (IQR)	3.0 (0.0–9.5)	2.0 (0.0–8.0)	0.0 (0.0–4.0)	1.8 (0.0–5.0)	0.33^a^
Informal care, week 3 (hours), median (IQR)	0.0 (0.0–4.3)	0.0 (0.0–5.0)	0.0 (0.0–4.0)	0.0 (0.0–2.0)	0.22^a^
Informal care, week 4 (hours), median (IQR)	0.0 (0.0–2.0)	0.0 (0.0–2.0)	0.0 (0.0–0.0)	0.0 (0.0–0.0)	0.04^a^
Informal care, week 5 (hours), median (IQR)	0.0 (0.0–0.0)	0.0 (0.0–0.0)	0.0 (0.0–0.0)	0.0 (0.0–0.0)	0.24^a^
Total time consumption for five weeks (hours), median (IQR)	12.5 (0.0–31.5)	11.0 (0.5–32.0)	10.0 (0.0–26.0)	7.0 (0.5–17.5)	0.23^a^
Summary of informal care in five weeks (hours)	2942.5	2878.0	3189.8	1780.5	–

*IQR* interquartile range; *SD* standard deviation; *TFU* telephone follow-up; *uTC* unplanned telephone contact; *uV* unplanned visit

^a^Kruskal–Wallis ANOVA; ^b^Mann–Whitney *U*-test; ^c^Comparison between Group C and Group D; ^d^Pearson’s Chi-squared test; ^e^Mean (SD)/median (IQR) number of uTCs in the group; ^f^Median (IQR) of the women who had uTCs; ^g^uVs either in primary healthcare, or in a hospital outpatient facility, or in both

The CPP for the nurse TFUs was set at a fixed average price per session, independent of the de facto time consumption. However, a significant difference was seen in time consumption between the groups at the first and second TFU. The group with only one TFU (Group B) had the highest time consumption on that occasion, but the coaching TFU (Group D) seemed to be more time consuming on these two occasions compared with Group C, whereas no differences were seen later.

The cost analysis is presented in Table 4. Since the duration of sick leave, and consequently the costs for sick leave, did not differ between the intervention groups, the indirect costs were made up of the informal care costs only in the cost analysis. The highest total cost per patient was seen in Group C with a cost of 2.4 times the total cost per patient in the group without TFUs (Group A). This was mainly attributed to the obviously higher costs for the TFUs in Group C and the higher costs of uVs. Compared with no use of TFU (Group A), the total cost per patient doubled when the TFUs included coaching (Group D). This was mainly caused by the higher costs for the TFUs in Group D, but the effect was counteracted by lower costs for fewer uTCs and lower costs for informal care in Group D.

**Table 4:** Accounting of costs for TFUs, uTCs, uVs, and informal care in relation to the intervention group

	Group A		Group B		Group C		Group D	
	(*n* = 120)		(*n* = 122)		(*n* = 125)		(*n* = 120)	
	No. of occasions or hours	€	No. of occasions or hours	€	No. of occasions or hours	€	No. of occasions or hours	€
Total cost of TFUs	0	0	122	9272	750	57,000	720	54,700
Total cost of uTCs	110	8360	135	10,260	109	8284	73	5548
Total cost of uVs	97	12,509	143	14,632	153	18,305	113	13,579
In primary healthcare								
Nurse	14	1064	62	4712	32	2432	18	1368
Physician	14	3066	4	876	16	3504	7	1533
In hospital care^a^								
Nurse	14	1064	21	1596	28	2128	18	1368
Gynecologist	55	7315	56	7448	77	10,241	70	9310
Costs of informal care in five weeks	2942.5 h	21,408	2878 h	21,009	3190 h	23,285	1780.5 h	12,998
Total cost of post-discharge contacts		42,349		55,173		106,874		86,845
Total mean cost per patient		353		452		855		724

*h* hours; *TFU* telephone follow-up; *uTC* unplanned telephone contact; *uV* unplanned visit

^a^hospital outpatient care

## Discussion

This study found that TFUs after benign hysterectomy seemed to be a cost-driving intervention without notable benefits for the women or the healthcare services. Originally, the study was planned to determine the cost-effectiveness of TFUs after benign hysterectomy but due to the absence of differences in recovery measures including HRQoL measurements between the four randomizations groups, we conducted a cost analysis [10, 11]. CMA is a suitable method to provide a health economical evaluation of a “new” intervention. This study demonstrated that implementation of TFU after hysterectomy consumed societal resources without improvement of the quality of care or HRQoL.

Choices between alternative interventions in healthcare are unavoidable. In contrast to everyday clinical decisions focusing on the individual patient, policy guidelines need to analyze a patient population and society from a broader perspective. Although qualitative studies have shown that women preferred nurse-led follow-up after surgery [7, 22], a health economic evaluation can aid in identifying the most advantageous option considering the limitations in the available healthcare resources.

The success of ERAS programs is often assessed by the duration of hospital stay. However, as emphasized by Kehlet and Joshi, this is only a surrogate marker of recovery [23]. In the perspective of having achieved shorter hospitalization times using ERAS programs, the programs could be extended to include factors that might influence the recovery after discharge from the hospital. Tailored TFU programs in different contexts may result in different outcomes. For instance, TFU of elderly patients after major gastro-intestinal surgery was associated with reduced length of postsurgical recovery [24].

When analyzing the different types of costs, it is obvious that TFU using structured coaching (Group D) had the lowest costs for uTCs as well as for informal care. Especially in comparison with Group C, which had the same number of planned TFUs, these findings indicate that the coaching content of the TFU program was of value. This finding seems to support the conclusion of Kersley Rydmark et al. in their qualitative study that empowerment to take control of the recovery process is a central theme in ERAS programs [25]. However, more studies are warranted to evaluate the impact of postoperative coaching follow-up models on recovery, especially in selected groups of vulnerable individuals at high risk of delayed recovery, and the health economic cost-effectiveness of the models.

A limitation of this study was the absence of qualitative questions addressing whether the women were pleased with their follow-up program. Moreover, the RNs were not specifically interviewed about their opinions on the TFU. A need to adapt the ERAS program to personal needs and individual goals has been identified from interviews [25]. It is possible that if sufficient information is provided to the women about postoperative recovery, in an ERAS setting, and the safety and assurance of contact possibility for questions and support can be ensured; then planned TFUs may be redundant.

CMA can be useful for healthcare decision-makers who need to allocate resources and make decisions and prioritize. However, it is important to remember that CMA is an instrument of economic evaluation, and other methods of analysis may be more appropriate depending on the specific context and goals of the analysis. The CMA in this study was conducted in a specific population and setting which may make it difficult to generalize the results. Another limitation of CMA is the sensitivity to changes in the price of interventions, which can have a significant impact on the results.

The shortage of nurses is today a fact in Sweden and in some other countries. It is necessary to use the nurses’ competence and working time effectively for the most cost-effective patient care. The nurses´ expenditure of time for TFU after benign hysterectomy can be replaced with time for other highly qualified tasks for nurses to improve the healthcare quality and the recovery of the patients. The unplanned contacts with a healthcare provider by telephone with a subsequent visit, if necessary, would perhaps be a more cost-effective alternative for women with complications after benign hysterectomy. However, to be successful such a strategy requires clear and easily accessible paths with instructions for the patient to make use of.

The results of this study should also be viewed in the perspective of advances in the application of patient-centered care and through the use of telemedicine. The development of telemedicine in healthcare over the past decade by introduction of health apps, online patient records, and virtual consultations appears to have improved patient health and reduced healthcare costs [26] and, possibly, indirectly to some extent contributed to addressing the shortage of nurses in healthcare. However, patients’ concerns about data security when using telemedicine have recently been noticed and appear to be an important factor to consider when offering telemedicine [27]. These factors should therefore be taken into consideration when offering telemedicine in a patient-centered healthcare system.

## Conclusion

The TFU models after benign hysterectomy used in this study seemed to be a cost-driving and inefficient way of using healthcare resources. However, the coaching TFU model appeared to both result in fewer uTCs and lower costs for informal care. The cost evaluation of TFUs and their impact on clinical outcomes must be considered before implementation of planned TFUs after surgery or other medical follow-ups.

## Data Availability

No datasets were generated or analysed during the current study.

## Abbreviations

CMA: Cost minimization analysis
CPP: Cost per patient
ERAS: Enhanced recovery after surgery
HRQoL: Health-related quality of life
TFU: Telephone follow-up
uTC: Unplanned telephone contact
uV: Unplanned visit
